# Polyacid Solutions as an Analogue of a Neural Network

**DOI:** 10.3390/polym18020279

**Published:** 2026-01-20

**Authors:** Sherniyaz Kabdushev, Dina Shaltykova, Eldar Kopishev, Gaini Seitenova, Rizagul Dyusssova, Ibragim Suleimenov

**Affiliations:** 1 Department of Chemistry and Technology of Organic Materials, Polymers and Natural Compounds, Al-Farabi Kazakh National University, Almaty 050040, Kazakhstan; sherniyaz.kabdushev.hw@gmail.com; 2 National Engineering Academy of the Republic of Kazakhstan, Almaty 050040, Kazakhstan; esenych@yandex.kz; 3 Department of Chemistry, L.N. Gumilyov Eurasian National University, Astana 010000, Kazakhstan; seitenova_gzh@enu.kz (G.S.); dyussova_rm@enu.kz (R.D.)

**Keywords:** polyacid, solution acidity, neural networks, hydrophilic interpolymer associates, neuromorphic materials, prebiological evolution, analogue of the Poisson-Boltzmann equation

## Abstract

Despite the increased interest in neuromorphic materials—a physical implementation of neural networks that could overcome the so-called von Neumann architecture’s limitations—most studies have been performed on the basis of systems specially constructed for this purpose. It has previously been shown that analogues of neural networks can spontaneously arise in solutions of hydrophilic polymers, but these systems involved molecules of different natures or required direct interaction between macromolecular clusters. The present paper proposes a theory that indicates the possibility of an analogue of neural network formation even in a single-component solution of a relatively weak polyacid. A model is suggested based on the account of heterogeneous distribution of polymer ionogenic groups within the volume leading to the fluctuations of electric fields and, as a result, to the local changes in the degree of ionisation of functional groups. Theoretical description of the system shows how it was reduced to a solution of the analogue based on the Poisson–Boltzmann equation. The results obtained showed that it is just fluctuations in the distribution of charges that provide the collective response of the system to external influences and serve as an argument in favour of analogy of such a solution within a neural network. The results are discussed in the context of a potential simple hydrophilic polymer system as a prototypical neuromorphic and evolving material that is relevant for organic electronics, metamaterials, and studies on prebiological evolution.

## 1. Introduction

Neuromorphic materials have attracted growing interest among researchers [1,2,3]. The literature reflects numerous attempts to implement such materials using a wide variety of chemical compounds [4,5,6], including those based on polymers [7,8]. In particular, attempts to implement neuromorphic materials based on polyelectrolyte hydrogels [9,10] are of interest.

As demonstrated in reviews [11,12], the issue of neuromorphic materials is currently considered mainly from the point of view of applied use. Neuromorphic materials are, in fact, the physical implementation of neural networks, which, as is well known, form the basis of the vast majority of existing artificial intelligence (AI) [13,14]. That is why neuromorphic materials, including those based on classical objects of polymer science, can be considered an important step towards the creation of next-generation AI [15,16], as well as next-generation computing systems, the need for which is dictated by objective reasons [17,18]. Current research in the field of neuromorphic materials is aimed at overcoming the fundamental shortcomings inherent in von Neumann architecture [19,20], and more broadly, this includes classical computing technology based on the use of binary logic. First and foremost, of course, this concerns the energy costs associated with the fact that in von Neumann architecture, the computing blocks and the blocks that store information are separated. It is assumed that computing systems based on neuromorphic materials will be free of this and similar shortcomings [19,20]. Neuromorphic materials capable of using, among other things, non-trivial algorithms [21] create real prerequisites for the implementation of AI that is close to its biological prototype: human intelligence. This statement is also methodologically justified because neuromorphic materials based, for example, on polymer hydrogels [22,23] are close to the biological prototype in terms of the chemistry of the processes used. The corresponding trends are also quite clear; they are associated, in particular, with work in the field of biocompatible neuromorphic materials [24,25] and organic-based electronics [26,27].

The use of system-based hydrophilic polymers, in which neural network analogues are formed spontaneously, provides additional prospects for the development of neuromorphic materials. (It should be noted that the existence of such systems was previously demonstrated in our earlier works [28,29]).

Namely, in works on nanotechnology carried out more than twenty years ago [30,31], a problem was clearly formulated that can be interpreted as the problem of creating tools that affect processes occurring at the molecular/supramolecular level. Numerous approaches based on self-organisation phenomena were proposed to solve this problem [32,33,34]. From a general methodological point of view, however, it is interesting to make the transition from self-organisation to tools based on the controlled evolution of the system used. Neural network analogues may in the future be used to ensure controlled evolution processes aimed, among other things, at obtaining neuromorphic materials with predetermined properties. The basis for this conclusion lies in the fact that algorithms enabling the formation of evolving neural networks have already been developed [35,36]. It should also be emphasised that demonstrating the formation of neural network analogues in polymer solutions is of interest from the standpoint of establishing general patterns in the evolution of complex systems [37,38]. The relevance of this issue is further connected to the elucidation of mechanisms underlying prebiological evolution [39,40].

Various perspectives exist within this field of research [41], including mutually exclusive ones [42]. An illustrative example is the concept proposed by Vanchurin [43], which is based on the findings of works [44,45]. This concept assumes that an evolving system represents an analogue of a neural network, thereby offering an alternative to all evolutionary theories that, in one way or another, trace back to Darwin’s concept. (These issues are discussed in detail in the Section 3).

However, the above-cited works [28,29] considered analogues of neural networks formed to be a result of interactions between molecules of different natures. Moreover, these works considered cases where macromolecular coils enter into direct interactions (hydrophobic, hydrogen bonds). Specifically, in [28] it was shown that hydrophobic–hydrophilic associates are formed in N-vinylpyrrolidone and vinylpropyl ether copolymer aqueous solutions, which can be considered analogous to neural networks (the interpretation of this term as applied to polymer solutions is presented in Section 2.1). The main interactions that determine the formation of such associates are hydrophobic interactions, which determine the nature of phase transitions in solutions of this type; the cited work presents a corresponding mathematical model. In work [29], the conclusion about the possibility of forming analogues of neural networks in polymer solutions is further confirmed, on the basis of considering associates stabilised by other types of interactions (hydrogen bonds, in particular). This work proposes a theory that shows that there is another type of interaction capable of leading to the formation of analogues of neural networks. This type of interaction manifests itself in solutions of relatively weak polyacids, and the formation of a neural network analogue is determined solely by the distribution of electrical charges.

## 2. Results

### 2.1. Analogues of Neural Networks: Interpretation of the Concept

**Figure 1:**
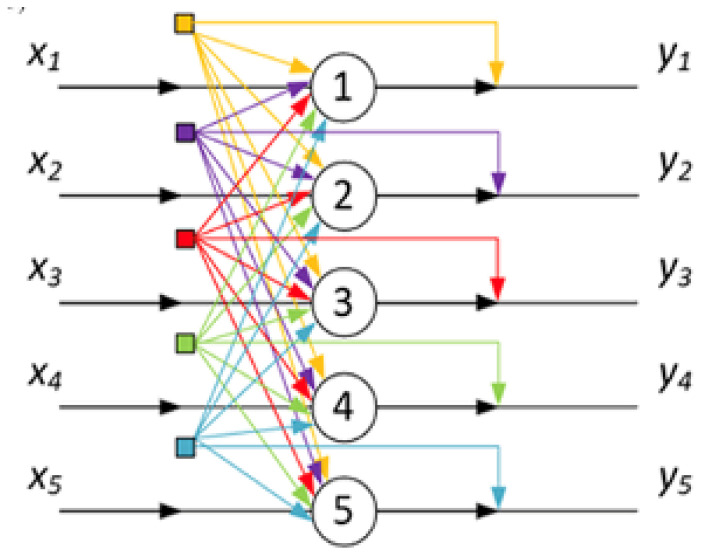
Hopf’s neural processor diagram (for five neurons [20]).

We start from the following formula, which describes a wide range of neural networks of various types, including Hopfield’s neural processor [36,46] (Figure 1),

(1) $$Y_{i}=Y_{i}\left(X_{i},Y_{1},Y_{2},\ldots ,Y_{N}\right)$$

where $Y_{i}$ is a variable describing the state of an individual neuron analogue, and $X_{i}$ is a variable describing the external influence on an individual neuron.

In applications, this formula is usually written using the neural network activation function $\sigma \left(X_{i},Y_{1},Y_{2},\ldots ,Y_{N}\right)$.

(2) $$Y_{i}=\sigma \left(X_{i},Y_{1},Y_{2},\ldots ,Y_{N}\right)$$

Formulas (1) and (2) are applicable not only to artificial neural networks of the Hopfield type, but also to a wide range of systems whose elements can interact with each other. Figure 1, among other things, clearly illustrates that the interaction of system elements with each other can be interpreted in terms of the formation of feedback loops between neuron analogues. This interaction is described by Formula (2) through the dependence of the activation function, including the values of variables $Y_{i}$, which describe the state of the system elements, which (based on the analogy with neural networks) is interpreted as the ‘output state’. The analogue of weight coefficients in this case is the degree of influence of the system elements on each other. This becomes particularly clear in cases where the influence of the system elements on one of them can be analysed within the framework of a linear approximation. Then Formula (2) takes the form that completely coincides with the formula describing the Hopfield neural processor:

(3) $$Y_{i}=\sigma (X_{i},\sum_{i\neq j}^{N}w_{ij}Y_{j})$$

where $w_{ij}$ are weight coefficients or their equivalents.

Formula (3) also allows us to clearly formulate the constraints imposed on the activation function. Namely, the activation function must not have extrema, and the values of $Y_{i}$ for any values of $X_{i}$ must lie within a limited interval. At the same time, the activation function, generally speaking, may experience discontinuities (for example, in cases when it is within the threshold). When these constraints are met, any system can be considered as an analogue of a Hopfield neural processor, and from the point of view of neural network theory, such systems will differ from each other only in the type of activation function.

Looking ahead a little, we note that if any of the parameters characterising the macromolecular tangle (e.g., its degree of swelling) are considered as variable $Y_{i}$, then these requirements are obviously met for a wide range of systems. Thus, the degree of swelling of the coil obviously varies from a minimum value (globule) to a maximum value. Exceptions are theoretically possible, but this implies that the degree of swelling of the macromolecular coil must depend non-monotonically on the controlling thermodynamic parameters.

The above considerations lead to the following formulation of the concept of a ‘neural network analogue’.

Any system whose behaviour is determined not only by external influences (e.g., the values of controlling thermodynamic parameters) but also by the mutual influence of its elements on each other can be considered an analogue of a neural network. From the point of view of mathematical description, this analogy corresponds to the fulfilment of Formula (2) with the above restrictions on the activation function or its analogue.

Note that with this formulation of the question, the specific ‘structure’ of the neural network or its analogue becomes secondary. The basis is precisely the mathematical description, but not the presence of specific elements (synapses, branching points, etc.).

When drawing analogies between complex systems of different natures and neural networks, however, a non-trivial question arises about the training of neural network analogues that arise, among other things, spontaneously [29,37]. The answer to this question can be given, among other things, from the point of view of the interpretation of the concept of a ‘complex system’ proposed in [38]. (This interpretation is based, among other things, on a general methodological consideration of the category of ‘complex’.) In accordance with the point of view reflected in the cited work, a complex system should be considered to be a system that is analogous to a neural network in the above sense.

The reasons for this are as follows. The most general considerations suggest that impacts on the system, depending on their nature, can be either ‘physical’ or ‘informational’ [38]. The terms are placed in quotation marks because they actually correspond to extreme cases (a purely physical impact is the Earth’s gravity, which determines the trajectory of a cannonball, while a purely informational impact is an order given by the leadership of a particular organisation). An impact acquires an ‘informational component’ if and only if a new quality (in the philosophical sense of the term) appears in it, specifically, the ability to process information. This quality is inherently present in neural networks. The simplest argument in favour of this point of view is the conclusion made in [37]: the state of a system that is converted into a neural network analogue depends not only on the current value of the control parameters (e.g., thermodynamic variables), but also on the specific law according to which these parameters changed at previous moments in time. It is this property that is the simplest manifestation of ‘memory’ possessed by many systems that fall into the category of complex in the above sense [37]. Consequently, when considering neural networks that arise spontaneously (more precisely, due to the specifics of systems, for example, those based on hydrophilic polymers [28,29]), the question of ‘learning’ should be interpreted as broadly as possible. ‘Learning’ is the result of previous (relative to the current) influences on the system. Such learning can be carried out in the classical sense (for artificial neural networks used in practice [37]). However, ‘training’—in the simplest case—can be interpreted through the dependence of the current state of the system on the nature of changes in control parameters at previous moments in time. An illustration of the existence of this kind of ‘memory’ as applied to systems of the type under consideration is presented in [38]. It was shown that even in the case of the simplest analogues of neural networks, the phenomenon of multidimensional hysteresis can be realised. However, it should be noted that even the simplest hysteresis phenomena observed in solutions of hydrophilic polymers [47] correspond to the interpretation of the term ‘learning’ under consideration. Indeed, in the presence of a hysteresis loop, as shown in the cited work, the current state of the system (polymer solution) depends not only on the current values of thermodynamic variables, but also on the nature of their change at previous moments in time. Thus, the analogy under consideration is valid from the point of view of the question of training neural networks or their analogues.

### 2.2. Prerequisites for Constructing a Theoretical Model

There are numerous theoretical works devoted to the study of polymer solutions, including polyacrylic acid [48,49,50], which is not surprising, since this object has been studied for a long time. A significant body of theoretical work presented in the literature; for example, [51,52,53], is based on the assumption that a polymer solution can be considered homogeneous (or close to it). In particular, in the cited works [51,52,53], the influence of the non-uniform distribution of functional groups throughout the polymer solution was not fully taken into account. The use of formal thermodynamics to describe the properties of polymer solutions is, of course, traditional in polymer science [54,55], but the possibilities of such theories are limited.

In particular, as shown in our works summarised in [37,38], this specificity consists, among other things, of the fact that there is a fairly wide range of conditions under which analogues of neural networks spontaneously form in polymer solutions (the term is used in the sense defined in Section 2.1). It is this factor that creates the prerequisites for the conversion of such systems into evolving objects.

The theory proposed in this work demonstrates that fluctuations in the distribution of ionogenic groups throughout the solution volume can significantly influence its properties and also lead to the formation of neural network analogues. This theory effectively shows that in solutions of relatively weak polyacids, another type of interaction (in addition to those considered in works [28,29]) can occur, which ensures the formation of a neural network analogue. We emphasise once again that it is possible to convert a neural network into an evolving system [37,38], which is of general interest both from the perspective of synergetics [56,57] and from the standpoint of the theory of evolution of complex systems [58,59] (these considerations are discussed in more detail in the Section 3).

The proposed theory is based on the following fact: in a polyacid solution, unlike in the case of a low-molecular-weight analogue, carboxyl groups are distributed unevenly throughout the volume. Their density reaches a maximum in the centres of the coils and decreases towards the periphery. This follows from the most fundamental models of polymer clusters (in particular, persistent [60,61]), according to which the distribution under consideration is close to Gaussian (experimental confirmation of the validity of such models has been repeatedly provided, including in works on the change in persistent length [62,63,64]). Additional arguments in favour of this conclusion also follow from the mathematical model proposed in [65].

Let us make some preliminary simple estimates, assuming that the macromolecular coils have finite sizes and the space between them is filled with a solution that does not contain polymer. Let us define c as the average concentration of acid groups R−H (both dissociated and undissociated) per total volume of the solution, i.e., c=N/V, where N is the total number of these groups and V is the volume of the solution. Let us define $c_{0}$ as the total concentration of acid groups within the model coil. Then the ratio

(4) $$\gamma =c_{0}/c\geq 1$$

can be considered as a measure of the heterogeneity of the solution. The higher γ, the higher the volume of the solution free of polymer.

Let us write down the dissociation reaction equation:

(5) $$R-H↔R^{-}+H^{+}$$

with a constant:

(6) $$K=\frac{\left[R^{-}\right]\left[H^{+}\right]}{\left[R-H\right]}$$

For relatively weak polyacids, we can assume that the degree of ionisation is relatively small, therefore

(7) $$\left[H^{+}\right]\approx \sqrt{Kc}$$

The last expression coincides with the estimate for the concentration of hydrogen ions in the solution, made under the assumption of a uniform distribution of functional groups.

Provided that these groups are concentrated within the coil, this estimate differs from (7) by a factor of $\sqrt{\gamma }$:

(8) $$\left[H^{+}\right]\approx \sqrt{Kc_{0}}=\sqrt{\gamma Kc}$$

The value (8) refers to the concentration of hydrogen ions within the coil. To obtain the average concentration (by solution volume), it must be divided by the ratio of the corresponding volumes, i.e., by γ. We have the following:

(9) $$\langle \left[H^{+}\right]\rangle \approx \frac{\sqrt{Kc}}{\sqrt{\gamma }}$$

This simple estimate shows that the expected value of the average proton concentration will be $\sqrt{\gamma }$ times less than that calculated under the assumption that the functional groups are distributed uniformly throughout the sample volume.

The higher the molecular weight, the higher the concentration of functional groups in the centre of the coil. This follows both from fundamental considerations of statistical physics of macromolecules [60,61] and from the model proposed in [65].

This simplest model shows only a completely obvious fact: the pH of a polyacid solution depends on the degree of its heterogeneity. However, despite the transparency of the conclusion made, it already allows us to substantiate the above statement: a polyacid solution is an example of a fluctuating system whose macroscopic characteristics depend on its structure.

Consequently, it is the nature of the fluctuations (at least at the level of macromolecular clusters) that should be the subject of research aimed at a more complete description of the properties of the solution (even if we do not yet take into account the neural network properties of the system under consideration).

We will show that the distribution of charges throughout the volume of the system with a non-uniform (and non-constant over time) distribution of acid group density is also important.

**Figure 2:**
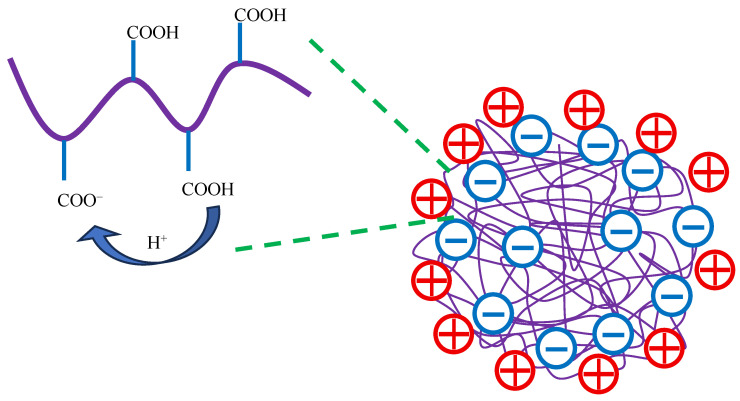
Mechanism of charge motion within the volume of the coil (**left**) and formation of an outer layer with increased ionisation (**right**).

Let us first consider an isolated coil formed by a relatively weak polyacid molecule. Only a small part of the carboxyl groups transforms into a charged form in this case, while degree of ionisation is small. At the same time, the charges formed during dissociation are by no means rigidly bound to a specific functional group; the charge is capable of moving throughout the volume of the system in the same way that ‘holes’ move throughout the volume of semiconductors, as illustrated in Figure 2.

More precisely, the movement of charge within the macromolecular coil is associated with the following effect. Under the influence of thermal motion, hydrogen ions tend to leave the coil. This is prevented by the attraction of negative charges carried by dissociated functional acid groups. At the same time, however, the outer positively charged layer affects the distribution of negative charges within the coil. Their concentration reaches a maximum at the periphery of the coil.

The increased charge concentration near the outer regions of the coil means, first of all, that its degree of swelling will be significantly higher than that predicted by theories based on the consideration of the polymer solution as a homogeneous phase. On this basis, it can be assumed that there is a critical concentration of coils at which they could occupy the entire volume of the solution completely. This conclusion is obviously true for hydrophilic polymers with a high degree of ionisation. It was previously made in [66] when considering alkali metal polyacrylates. In [66], it was shown that there are conditions under which the swelling of polyelectrolyte coils is limited by contacts with neighbouring ones, and the solution as a whole represents a kind of mosaic in which the coils almost completely fill the volume of the solution.

It is convenient to estimate the critical concentration of clusters based on an easily measurable value: the degree of swelling of a cross-linked network of the same chemical composition. Namely, it can be assumed that the critical concentration (the coils already fill the entire volume but do not yet prevent each other from swelling) approximately corresponds to the concentration of the hydrophilic polymer in an equilibrium-swollen gel with a sufficiently low cross-link density. Typical values of the degree of swelling for sodium polyarylate-based gels are 300–400 g of water per 1 g of dry matter [67,68], which corresponds to a concentration of this polymer of 0.25–0.3 g/litre or 0.035–0.045 mol L^−1^.

This assessment applies to strong polyelectrolytes. Consequently, it is reasonable to conclude that the concentration of functional groups of weak polyelectrolytes is unevenly distributed throughout the solution (all other conditions being equal).

### 2.3. Theory of Fluctuating Electrostatic Interactions

The prerequisites for constructing the theory are as follows. First, the coil is a loose, highly fluctuating system. Second, as follows from the materials of works [28,29], there are conditions when structures analogous to neural networks (the term is used in the sense specified in Section 2.1) are formed in the polymer solution: hydrophilic and hydrophobic–hydrophilic associates.

**Figure 3:**
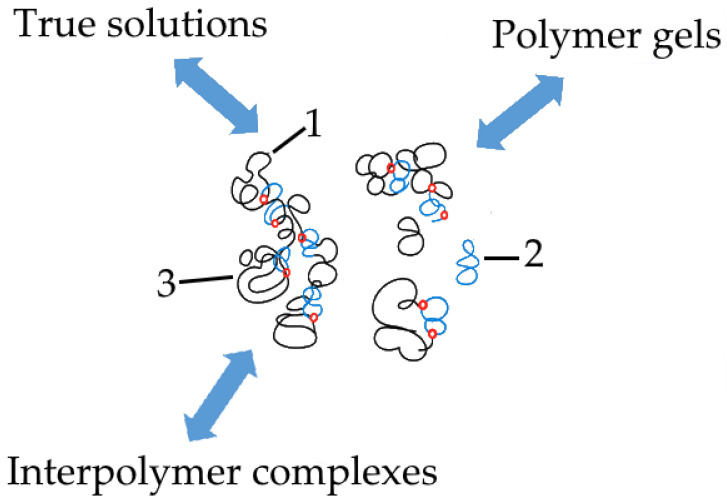
A dynamic interpolymer network (central part of the figure) as a non-trivial object of study in polymer science; 1, 2—interacting coils of different nature; 3—regions of formation of unstable bonds between the coils.

Such structures exist in a dynamic mode. In this mode, the bonds between macromolecular coils are continuously broken and re-formed. A diagram reflecting the position of the structures under consideration in the classification of objects formed as a result of interactions between macromolecules in solution is shown in Figure 3. We emphasise that this diagram was constructed by taking into account the results we obtained earlier [28,29,69].

The specificity of the system considered in this work is determined mainly by fluctuations in the distribution of acid groups throughout the solution volume. These fluctuations give rise to heterogeneous electric fields, which, in turn, directly affect the degree of ionisation of the functional groups. It is important to note that the variations in the degree of ionisation caused by this factor occur locally, providing additional evidence for the validity of the analogy between the solution of this type and a neural network. Indeed, local transformations in the properties of the solution can be regarded as analogous to changes in the states of neurons that constitute a neural network. In other words, our proposed theory aims to show that a system similar to the one shown in the centre of Figure 3 can only arise as a result of the uneven distribution of electric charges throughout the volume of the solution.

The emergence of pronounced local fluctuations of electric fields in the solution under consideration is demonstrated below based on an analysis of the system of equations, which allows us to find the distribution of electric fields (or potentials). This system of equations includes the following:

1. Poisson’s equation, which relates the electrostatic potential φ and the distribution of electric charges ρ:

(10) $$\rho =-\varepsilon_{0}\varepsilon \nabla^{2}\varphi$$

where

(11) $$\rho =e\left(\left[H^{+}\right]-\left[R^{-}\right]\right)$$

2. Chemical kinetics equations that take into account the non-uniform distribution of carboxyl groups in space:

(12) $$\frac{dc^{+}}{dt}=-D\nabla^{2}c^{+}+be\nabla (\vec{E}c^{+})+k_{1}c_{0}-k_{2}c^{+}c^{-}$$

(13) $$\frac{dc^{-}}{dt}=k_{1}c_{0}-k_{2}c^{+}c^{-}$$

where $c^{+}=\left[H^{+}\right]$, $c^{-}=\left[R^{-}\right]$, D and b—the diffusion and mobility coefficients of hydrogen ions, respectively, $\vec{E}=-\nabla \varphi$—the electric field intensity vector, e—the elementary charge, $k_{1}$ и $k_{2}$—the reaction rate constants for the dissociation of the carboxyl group and the reverse reaction, ε и $\varepsilon_{0}$—the dielectric permittivities of the medium and vacuum.

The system should also include an equation for the change in the concentration of undissociated R−H groups, but at low degrees of polymer ionisation, this concentration can be considered constant and equal to $c_{0}$.

Equation (12) can be analysed within the framework of the quasi-stationary approximation. Indeed, the rate of restructuring of the electric field in the system under consideration is determined by the velocity of hydrogen ions, while the rate of change in macromolecular clusters in solution is determined by the rates of change in the shape and mutual arrangement of the clusters themselves.

Therefore, it is permissible to assume that all time derivatives are zero. In this case, it follows from Equations (8) and (9) that

(14) $${\left(c^{+}\right)}^{2}+c^{+}\frac{\varepsilon_{0}\varepsilon }{e}\nabla^{2}\varphi -Kc_{0}=0$$

where

(15) $$K=\frac{k_{1}}{k_{2}}$$

Equation (14) allows us to express the concentration of mobile ions through the field distribution and the distribution of functional group concentrations:

(16) $$c^{+}=-\frac{1}{2}\frac{\varepsilon_{0}\varepsilon }{e}\nabla^{2}\varphi \pm \frac{1}{2}\sqrt{4Kc_{0}+{\left(\frac{\varepsilon_{0}\varepsilon }{e}\nabla^{2}\varphi \right)}^{2}}$$

The value $\frac{1}{2}\frac{\varepsilon_{0}\varepsilon }{e}\nabla^{2}\varphi$ can be interpreted as the deviation of the concentration c^+^ from the average concentration of charged particles $\frac{1}{2}\left(c^{+}+c^{-}\right)$. Indeed, as follows from (11) and (16), the concentration of dissociated carboxyl groups can be expressed as follows:

(17) $$c^{-}=\frac{1}{2}\frac{\varepsilon_{0}\varepsilon }{e}\nabla^{2}\varphi \pm \frac{1}{2}\sqrt{4Kc_{0}+{\left(\frac{\varepsilon_{0}\varepsilon }{e}\nabla^{2}\varphi \right)}^{2}}$$

This expression differs from Formula (16) only in the sign of the linear term. Consequently, the average concentration of mobile particles is as follows:

(18) $$\frac{1}{2}\left(c^{+}+c^{-}\right)=\pm \frac{1}{2}\sqrt{4Kc_{0}+{\left(\frac{\varepsilon_{0}\varepsilon }{e}\nabla^{2}\varphi \right)}^{2}}$$

From Formula (18), in particular, it follows that a positive sign should be chosen for the square root in all the expressions given above.

We also emphasise that, for physical reasons, it is appropriate to consider the average concentration $\frac{1}{2}\left(c^{+}+c^{-}\right)$ as a quantity that reflects, among other things, the movement of charges to the edge of the coil, as illustrated in Figure 2. We emphasise once again that, due to the attraction of opposite electrostatic charges, such a displacement is carried out in a self-consistent manner.

It is clear from Formula (18) that any inhomogeneities arising in the system under consideration obviously affect the degree of ionisation of acid groups. This influence depends on the square of the electric field gradient. This means that the more pronounced the inhomogeneities are, the greater the effective ionisation of the acid groups will be. This indicator will reach its maximum value in the vicinity of the double electric layer analogue shown in Figure 2. Physically, this conclusion is extremely clear. In those areas of the solution where double electric layer analogues are realised (more precisely, where there is a spatial displacement of the cloud of positive charges relative to the distribution of negative charges), the reaction rate, which is the inverse of the dissociation reaction of carboxyl groups, decreases. Thus, as a result of the heterogeneous distribution of electric fields in the solution of the considered type, regions with pronounced variations in the concentration of charged particles indeed arise, which, as noted above, serves as an argument in favour of the analogy between such a solution and a neural network.

Let us show that the theoretical description of the distribution of electrostatic fields in the system under consideration must obey an analogue of the Poisson–Boltzmann equation, which is widely used in modern physical chemistry of polyelectrolytes. It should be recalled that the Poisson–Boltzmann equation is derived on the basis of the following principles: the spatial distribution of the electrostatic potential is governed by the equations of electrostatics (specifically, by the Poisson equation), whereas the dependence of the concentration of charged species on the electrostatic potential is determined by the Boltzmann distribution.

It is important to note that the Boltzmann distribution formula can be derived not only from thermodynamic considerations but also directly from the equation of motion for charged particles. Indeed, in the stationary case, Equation (12) takes the form:

(19) $$-D\nabla^{2}c^{+}+be\nabla \left(\vec{E}c^{+}\right)=0$$

The solution to this equation, as is well known, is the Boltzmann distribution:

(20) $$c^{+}=c_{0}^{+}\exp\left(-\frac{e\varphi }{kT}\right)$$

where $c_{0}^{+}$—concentration of positively charged ions at a point φ=0.

This result also corresponds to well known thermodynamic considerations. Consequently, the equation for the distribution of the electrostatic field in the system under consideration can be obtained by equating solution (20) to the solution that follows from Formula (15). We have the following:

(21) $$c_{0}^{+}\exp\left(-\frac{e\varphi }{kT}\right)=-\frac{1}{2}\frac{\varepsilon_{0}\varepsilon }{e}\nabla^{2}\varphi +\frac{1}{2}\sqrt{4Kc_{0}+{\left(\frac{\varepsilon_{0}\varepsilon }{e}\nabla^{2}\varphi \right)}^{2}}$$

The limiting case corresponding to $\nabla^{2}\varphi =0$, φ=0 gives a result that coincides with that obtained from elementary considerations. Namely, in this case, as expected, we have the following:

(22) $$c_{0}^{+}=\sqrt{Kc_{0}}$$

Equation (21) can be transformed as follows:

(23) $$Z_{0}\exp\left(-\Phi \right)=-\frac{1}{2}\lambda^{2}\nabla^{2}\Phi +\sqrt{1+{\left(\frac{1}{2}\lambda^{2}\nabla^{2}\Phi \right)}^{2}}$$

where $\Phi =\frac{e\varphi }{kT}$—reduced dimensionless potential, $Z_{0}^{+}=\frac{c_{0}^{+}}{\sqrt{Kc_{0}}}$—reduced dimensionless concentration of positive ions, λ—Debye length.

(24) $$\lambda^{2}=\frac{\varepsilon_{0}\varepsilon kT}{e^{2}\sqrt{Kc_{0}}}$$

From Equation (23) it follows that

(25) $$-\lambda^{2}\nabla^{2}\Phi =Z_{0}^{+}\exp\left(-\Phi \right)-{\left(Z_{0}^{+}\right)}^{-1}\exp\left(\Phi \right)$$

Equation (25) can be considered analogous to the Poisson–Boltzmann equation. The difference is that the second term on the right-hand side describes not the distribution of mobile negative charges, but the charges arising from the dissociation of functional groups, i.e., this term corresponds to the solution of Equation (13) for the stationary case:

(26) $$c^{-}c^{+}=Kc_{0}$$

From which

(27) $$Z_{0}^{+}Z_{0}^{-}=1$$

where $Z_{0}^{-}=\frac{c_{0}^{-}}{\sqrt{Kc_{0}}}$.

**Figure 4:**
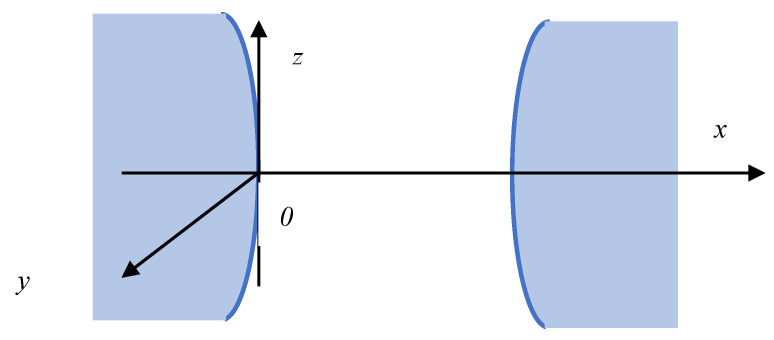
Choosing a coordinate system for derivation of Equation (26).

Let us further consider a locally flat section of a macromolecular tangle, which may be located at some distance from its neighbour (Figure 4). In doing so, we will select the *0x* axis perpendicular to the surfaces under consideration. We will measure the potential from the origin located on the surface of the local section of the macromolecular tangle.

Then, Equation (25) becomes a second-order ordinary differential equation.

(28) $$-\lambda^{2}\frac{d^{2}\Phi }{{dx}^{2}}=Z_{0}^{+}\exp\left(-\Phi \right)-{\left(Z_{0}^{+}\right)}^{-1}\exp\left(\Phi \right)$$

A similar equation can be immediately written for the solution (water) sphere, which is precisely the Poisson–Boltzmann equation.

(29) $$-\lambda^{2}\frac{d^{2}\Phi }{{dx}^{2}}=Z_{0}^{+}\exp\left(-\Phi \right)$$

We emphasise that, given the chosen origin of coordinates, the value $Z_{0}^{+}$ represents the concentration of positive ions at the boundary of the coil.

Equations (28) and (29) allow for an analytical solution, but there is no need to find it yet. The main relationships can be obtained based on a direct analysis of the first integrals of these equations.

Let us consider the following expression.

(30) $$P_{1}=\int_{-\infty }^{0}\rho \left(x\right)E\left(x\right)dx$$

The value $P_{1}$ can be interpreted [70] as a component of the swelling pressure of the coil caused by the polyelectrolyte effect. Indeed, the integral (30) is numerically equal to the force acting on the uncompensated charges of the coil from the electric field, relative to a unit area. A similar expression can be written for uncompensated charges in the solution near the surface of the coil.

(31) $$P_{2}=-\int_{0}^{\infty }\rho \left(x\right)E\left(x\right)dx$$

The electric fields outside and inside the coil are directed in opposite directions, which is taken into account by the minus sign in Formula (31). Taking the sign into account, the balance is expressed by the following formula.

(32) $$P_{1}=P_{2}$$

Expressing the distribution of charge and electric field through potential derivatives, the balance condition (32) can be rewritten in the following form.

(33) $$-\int_{-\infty }^{0}\frac{d\Phi }{dx}\frac{d^{2}\Phi }{{dx}^{2}}dx=\int_{0}^{\infty }\frac{d\Phi }{dx}\frac{d^{2}\Phi }{{dx}^{2}}dx$$

Both parts of Formula (33) are transformed as follows:

(34) $$\lambda^{2}\int \frac{d\Phi }{dx}\frac{d^{2}\Phi }{{dx}^{2}}dx=\frac{1}{2}\lambda^{2}{\left(\frac{d\Phi }{dx}\right)}^{2}$$

Multiplying Equations (28) and (29) by $\frac{d\Phi }{dx}$ and integrating over the coordinate, we obtain

(35) $$\frac{1}{2}\lambda^{2}{\left(\frac{d\Phi }{dx}\right)}^{2}=Z_{0}^{+}\exp\left(-\Phi \right)+{\left(Z_{0}^{+}\right)}^{-1}\exp\left(\Phi \right)-C_{10}$$

(36) $$\frac{1}{2}\lambda^{2}{\left(\frac{d\Phi }{dx}\right)}^{2}=Z_{0}^{+}\exp\left(-\Phi \right)-C_{20}$$

Let us assume that the sizes of the coil are at least several times greater than the Debye length. Then, based on physical considerations, we can conclude that in an area sufficiently distant from the coil boundary, the electric field becomes zero. Let us denote the potential in such an area by $\Phi_{-\infty }$. Then,

(37) $$\begin{array}{c} \frac{1}{2}\lambda^{2}{\left(\frac{d\Phi }{dx}\right)}^{2}=Z_{0}^{+}\exp\left(-\Phi \right)+{\left(Z_{0}^{+}\right)}^{-1}\exp\left(\Phi \right)-Z_{0}^{+}\exp\left(-\Phi_{-\infty }\right)\\ -{\left(Z_{0}^{+}\right)}^{-1}\exp\left(\Phi_{-\infty }\right) \end{array}$$

If the electric field in the area under consideration is zero, then the medium in it must be electrically neutral, i.e., the concentrations of positive and negative charges must be equal. We have

(38) $$Z_{0}^{+}\exp\left(-\Phi_{-\infty }\right)={\left(Z_{0}^{+}\right)}^{-1}\exp\left(\Phi_{-\infty }\right)$$

Equation (38) can be rewritten as

(39) $$\exp\left(-\Phi_{-\infty }+\ln Z_{0}^{+}\right)=\exp\left(\Phi_{-\infty }-\ln Z_{0}^{+}\right)$$

from which

(40) $$\Phi_{-\infty }=\ln Z_{0}^{+}$$

Consequently, Equation (37) for the square of the reduced electric field inside the coil can be rewritten as follows:

(41) $$\frac{1}{2}\lambda^{2}{\left(\frac{d\Phi }{dx}\right)}^{2}=Z_{0}^{+}\exp\left(-\Phi \right)+{\left(Z_{0}^{+}\right)}^{-1}\exp\left(\Phi \right)-2$$

Once the origin has been chosen (point 0 is located on the boundary of the tangle), it follows from relation (41) that

(42) $${\left.\frac{1}{2}\lambda^{2}{\left(\frac{d\Phi }{dx}\right)}^{2}\right|}_{-0}=Z_{0}^{+}+{\left(Z_{0}^{+}\right)}^{-1}-2$$

Based on similar considerations, we can examine the behaviour of the electric field outside the coil. Provided that the coils are sufficiently far apart (by several Debye lengths), we can also specify the area in which the electric field is zero. Denoting the potential in this area by $\Phi_{\infty }$, we obtain

(43) $$\frac{1}{2}\lambda^{2}{\left(\frac{d\Phi }{dx}\right)}^{2}=Z_{0}^{+}\exp\left(-\Phi \right)-Z_{0}^{+}\exp\left(-\Phi_{\infty }\right)$$

Recall that we are considering a solution containing only polyacid. Consequently, the proton concentration away from the coil is determined solely by water dissociation, i.e., the value $Z_{0}^{+}\exp\left(-\Phi_{\infty }\right)$—the reduced proton concentration in the area away from the coil can be considered negligible. Then,

(44) $${\left.\frac{1}{2}\lambda^{2}{\left(\frac{d\Phi }{dx}\right)}^{2}\right|}_{0}=Z_{0}^{+}$$

The left-hand sides of Equations (42) and (44) must be equal, which satisfies the condition of continuity of the electric field at the boundary between the droplet and the solution. Equating them, we obtain

(45) $$Z_{0}^{+}=\frac{1}{2};\;Z_{0}^{-}=2$$

**Figure 5:**
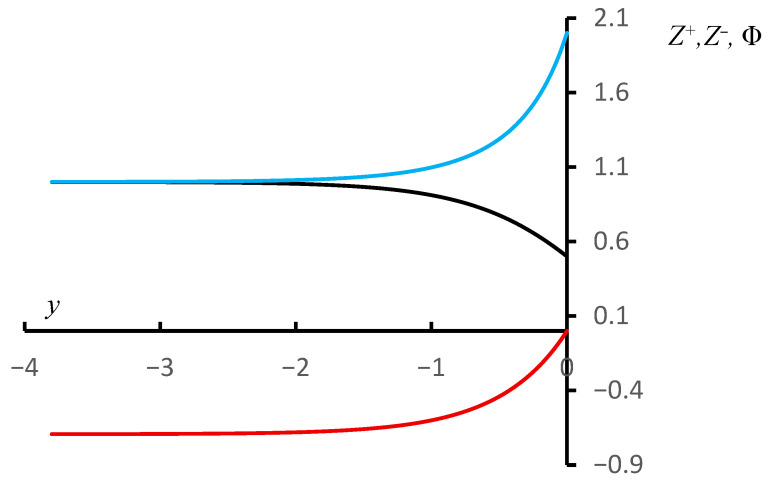
Profiles of reduced concentrations of negative (blue) and positive (black) charges and reduced potential (red) in the surface layer of a model macromolecular coil.

This result appears quite straightforward. However, it confirms the point of view expressed earlier on the basis of qualitative considerations. Negative charges formed as a result of partial dissociation of acid groups behave like ‘holes’ in a semiconductor, i.e., they move through the volume of the coil. Moreover, they actually demonstrate the effect of mutual repulsion, as a result of which the density of the negative charge at the boundary of a homogeneous (model) coil is twice as high as in its volume. The profiles of the reduced concentrations and reduced potential for this case are shown in Figure 5. The graphs presented in this figure are based on accurate analytical solutions, which are discussed in Supplementary Materials. The abscissa axis in this figure shows the reduced dimensionless coordinate $y=\frac{x}{\sqrt{2}\lambda }$. It can be seen that, as expected, deviations from constant values occur only within a distance of about 2–3 Debye lengths from the surface, which corresponds to the assumption used. It can also be seen that the solution corresponds to the qualitative model illustrated in Figure 2: negative charges behave like ‘holes’ in a semiconductor, accumulating near the surface of the coil.

The generalisation of Formula (44) can be written in the following form

(46) $${\left.\frac{1}{2}\lambda^{2}{\left(\frac{d\Phi }{dx}\right)}^{2}\right|}_{0}=\left(1-\mu \right)Z_{0}^{+}$$

where μ—is a parameter equal to the ratio of the proton concentration at the point in the solution where it reaches its minimum to the proton concentration at the boundary of the model coil, i.e., 0≤μ<1. For the extreme case considered above, it is obvious that μ=0. The situation where μ≠0 occurs when the coils approach each other at a sufficiently close distance. In this case, the condition of continuity of the electric field at the boundary of the coil implies that

(47) $$\left(1-\mu \right)Z_{0}^{+}=Z_{0}^{+}+{\left(Z_{0}^{+}\right)}^{-1}-2$$

Or in terms of the reduced concentration of negative charges

(48) $${\left(Z_{0}^{-}\right)}^{2}-2Z_{0}^{-}+\mu =0$$

**Figure 6:**
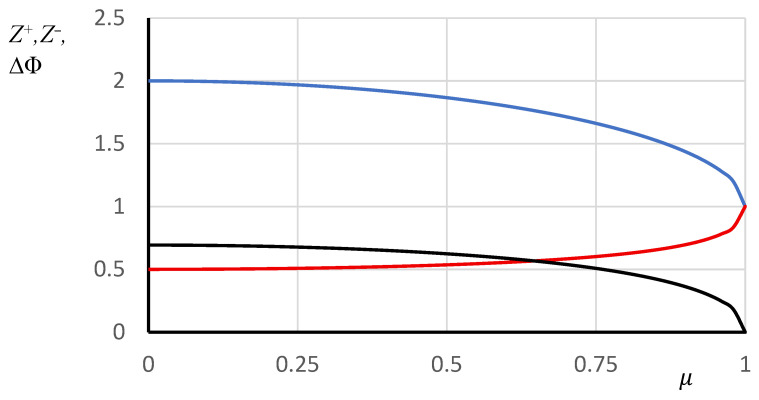
Dependencies of the reduced concentrations of negative (blue) and positive (red) charges and the reduced potential (black) in the surface layer of a model macromolecular coil on the parameter.

The solution to this equation is elementary: $Z_{0}^{-}=1+\sqrt{1-\mu^{2}}$; its graph, as well as the dependencies of the reduced proton concentration and reduced potential on the parameter μ are shown in Figure 6.

The proposed theory shows, first of all, that specific interactions may occur between macromolecular coils of a relatively weak polyacid, associated with a change in the characteristics of surface double layers when the coils come into contact with each other. Indeed, as follows from Equations (27) and (32), the degree of swelling of a coil depends, among other things, on the density of the surface double layer. In the absence of contact, the limiting value of the concentration of negative charges is twice their concentration in the volume of the coil, but this difference is significantly reduced upon contact (Figure 6). Consequently, contact between coils leads to a change in their degree of swelling.

**Figure 7:**
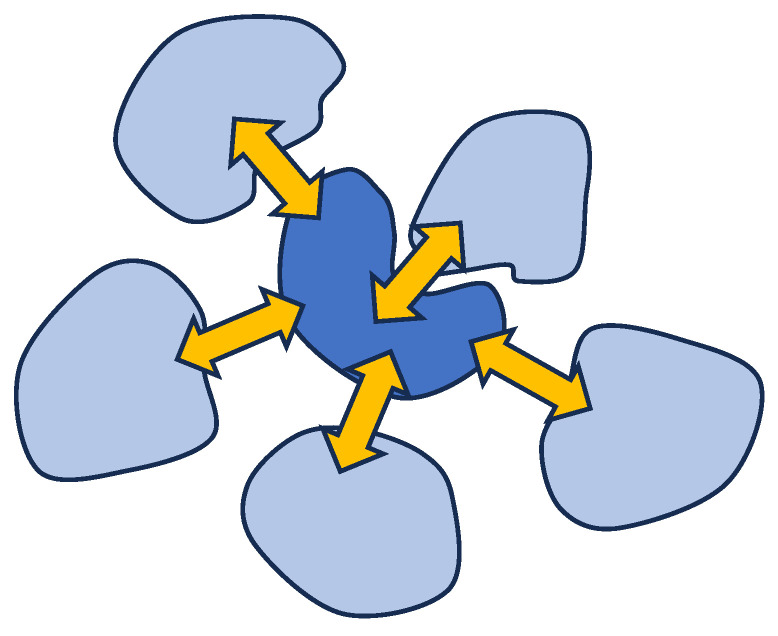
Illustration of the contact interaction of weak polyacid coils in solution.

Taking into account the arguments presented in [28,29], it can be concluded that a solution containing only molecules of a relatively weak polyacid also forms an analogue of a neural network, as illustrated in Figure 7.

This figure is similar to the one used in [29,37], demonstrating that the presence of interactions between neighbours already converts the system under consideration into a direct analogue of Hopfield’s neural processor. It can be seen that the feedback system that arises in a system whose elements interact with each other is topologically equivalent to the Hopfield neural network diagram shown in Figure 1. Figure 7 emphasises that the state of an individual coil depends, among other things, on the nature of its interaction with neighbouring coils.

## 3. Discussion

Thus, specific (contact) interactions may occur between relatively weak polyacid coils due to the nature of the formation of surface double electric layers (which, in turn, is due to the uneven distribution of the degree of ionisation of acid groups). Consequently, it can be argued that a dynamic network structure similar to that considered in [28,29] is indeed formed in the system under consideration. The difference is that this work proves that such a structure is formed even when hydrophobic interactions do not participate in the formation of a neural network analogue, hydrogen bonds are not formed, etc. This is expressed, in particular, in the fact that the reaction of a system of this type to any external influences will be collective in nature, since a change in the state of a single macromolecular coil will cause a change in the state of neighbouring ones [29]. It should also be noted that this conclusion can be extended to polyelectrolyte objects of a different nature (e.g., nanogels or compositions based on them).

A comparison of the main results reported in previously published studies with those obtained in the present study is presented in Table 1.

It should also be noted that the results obtained allow for a natural generalisation in the case where polymer solutions containing low-molecular-weight salts or other low-molecular-weight compounds capable of affecting the acidity of the solution are considered. It is easy to show that an analogue of Equation (28) can be derived in the general case, i.e., when ions of different types are present in the system. The generalised equation has the form

(49) $$-\lambda^{2}\frac{d^{2}\Phi }{{dx}^{2}}=Z_{0}^{+}\exp\left(-\Phi \right)+\sum_{i=1}^{M}Z_{i0}q_{i}\exp\left(-q_{i}\Phi \right)-{\left(Z_{0}^{+}\right)}^{-1}\exp\left(\Phi \right)$$

where $Z_{i0}$ is the concentration of ions of type i at the point where Φ=0, $q_{i}$ is the charge number of ions of the specified type (taking into account the sign), and the Debye length is calculated using the standard formula, taking into account the presence of several types of ions in the solution.

Formula (49) emphasises that, regardless of the presence of additional ions in the system under consideration, the distribution of the intrinsic charges of the macromolecular coil also obeys an analogue of the Boltzmann distribution. This means, among other things, that the main conclusion confirmed by the calculations presented in Figure 5 remains valid. The distribution of the intrinsic charges of the coil (i.e., the charges of dissociated acid groups) is formed by a mechanism analogous to the movement of ‘holes’ in semiconductors. The mutual repulsion of such charges leads to an increase in their concentration at the periphery of the coil. This factor remains significant as long as the obvious condition is met: the concentration of hydrogen ions must be at least comparable to the concentration of other types of low-molecular ions. Otherwise, the formation of double layers will proceed according to the mechanism described in [70], i.e., ultimately determined by the classical Donnan equilibrium.

Taking into account the results of works [35,38], it can be concluded that even the simplest—and seemingly well-studied—systems based on water-soluble polymers can be used to create evolving systems built on analogies with neural networks (in the sense reflected in Section 2.1). It is possible that in this regard, it will also be necessary to rethink a significant amount of experimental data reflected in the literature and devoted, in particular, to the interactions of carboxylic acids with salts of polyvalent metals [71,72,73]. Specifically, the results of this work show that in this case there is an interaction between a solution containing polyvalent ions and an object with a rather complex structure (albeit dynamic). Ions capable of forming two or more valence bonds can obviously not only form an insoluble polysalt but also affect the structure under consideration as a whole, acting as a kind of cross-agent.

This issue is of interest, among other things, from the point of view of the creation of organic electronics, which is currently undergoing active development, and which is focused, among other things, on the use of conductive media with different types of conductivity [74,75]. In the future, technologies may be implemented in which such systems are formed through controlled evolution processes.

Solving problems of this kind is, of course, a rather distant prospect, but there are a number of pressing issues for which the ability to artificially create inhomogeneities in a controlled manner is relevant. This applies, for example, to the creation of metamaterials [76,77], which are of interest, for example, for ensuring the radar invisibility of aircraft [78,79]. Metamaterials are usually dielectric (e.g., polymer) matrices containing conductive inclusions of various shapes [80,81,82]. Currently, metamaterials are manufactured using metal parts, but this is not mandatory. In the future, conductive inclusions in a polymer matrix formed as a result of its interaction with polyvalent metal salts may also be used for this purpose. Taking into account the results obtained in organic electronics [83,84,85], in this case it is permissible to raise the question of combining the ability of neuromorphic material to process information with the effect that the result of such processing has on the material itself. This approach seems particularly important at this stage, since the introduction of neuromorphic materials into practice must obviously be focused on fairly simple systems that perform only a limited number of operations. Corresponding trends can also be observed in practice [86,87].

In the future, there will be quite broad opportunities related to the control of the simplest types of evolutionary processes aimed at creating structures with a given type of order (including conductivity). Such influences can be active (it is known that electric current has a significant effect on the behaviour of polyelectrolytes [88,89,90]), and there are also polymer-based systems that are sensitive to magnetic fields [91,92] and optical radiation [93,94]), or based on the use of additional reagents that ensure, for example, the formation of interpolymer complexes. Such complexes have also been well-studied [95,96,97,98], and it has been shown that they are also suitable for the formation of nanoscale objects [99,100].

The formation of complexes creates additional opportunities: as noted in [38], the neural network properties of physicochemical systems of the type under consideration become pronounced due to the use of remote interaction effects between macromolecules that are proton donors and acceptors [101,102,103]. Any weak polyacid, including polyacrylic acid, can act as a proton donor in this type of interaction. Any hydrophilic polymers containing, for example, amine functional groups can act as proton acceptors. The modernisation of the system under consideration can, accordingly, be achieved by forming complexes that simultaneously perform the functions of proton acceptors. In general, it can be argued that there is a wide range of effects on neural network analogues that spontaneously form even in solutions of well-studied polymers.

The results obtained in this work are also of methodological interest (taking into account the conclusions made in [37]).

The evidence that neural network analogues can spontaneously form in polymer solutions (including those containing a single component) is of interest from the standpoint of elucidating the mechanisms of prebiological evolution [39]. In essence, this is a fundamentally important question (from the point of view of the neural network concept of evolution): could an analogue of a neural network arise in an environment that at first glance appears to be purely homogeneous?

It should be noted that the foundations of neural network theory of evolution were laid in the works of V. Vanchurin and co-authors [43,44,45]. Namely, in [43], based on the results obtained in [44,45], a rather unusual hypothesis was put forward, according to which the Universe as a whole can be viewed through an analogy with a neural network. In the cited works, analogies with neural networks were used to establish the mechanisms of evolution preceding biological evolution. As is well known [39], this problem remains unresolved, despite considerable efforts. It is worth noting that Vanchurin’s hypothesis can be considered as an alternative to the Darwinian point of view (as well as its various variants [40,41,42,43]), since, according to this hypothesis, the mechanism of evolution of a complex system is determined not only and not so much by fluctuations (mutations) in the characteristics of its elements, but rather by systemic properties. A similar point of view was previously expressed in [104].

This hypothesis has not yet gained widespread recognition, although there are many arguments of a general methodological and even philosophical nature in its favour [38]. These arguments are related, among other things, to very specific results obtained, in particular, in works [105,106], and relating to complex systems of various types (the term ‘complex systems’ is understood in accordance with work [38]). Thus, in work [105], it was shown that any voting council can be considered analogous to a neural network. In works [106,107], a neural network model of society was substantiated, etc. In works [28,29], it was shown that analogues of neural networks can be formed in polymer solutions.

The results presented in this work, once again demonstrating that complex systems (in particular, weak acid solutions) can be converted into analogues of neural networks, serve as another argument in favour of V. Vanchurin’s concept. Indeed, this concept, as formulated in [43], cannot be falsified or experimentally verified, since the object of study in the cited work is the universe as a whole. From this point of view, simpler systems that correspond to this concept but nevertheless allow for direct experimental study are of interest. From our point of view, such systems are solutions of hydrophilic polymers. At a minimum, they can serve as a model for studying the processes that allowed such sophisticated information processing systems as living organisms to emerge. The main argument in favour of this point of view is as follows. Solutions of hydrophilic polymers are capable of converting into an analogue of a neural network, i.e., they acquire the ability to process information. These systems are capable of evolving, i.e., they can give rise to more complex information processing systems, in accordance with the conclusions of [37]. At the same time, the nature of evolution, in accordance with the concept [43], is determined not by random factors (mutations), but by the properties of the system as a whole, which also corresponds to a point of view alternative to Darwinism.

The most advanced information processing system known to date (the human brain) is also a neural network that is built on the basis of hydrophilic polymers. Consequently, from a general methodological point of view, the task boils down to finding the missing evolutionary links between the simplest types of neural networks and the most sophisticated of the known reliable variants (we emphasise that, from a methodological point of view, these links fully correlate with the nature of the above-mentioned limiting cases).

This thesis provides sufficient grounds for more in-depth research into the neural network properties of hydrophilic polymers.

## 4. Conclusions

This work presents theoretical evidence that aqueous solutions of weak polyacids, such as polyacrylic acid, can self-create dynamic network structures, similar to neural networks, even in the complete absence of hydrophobic interactions, hydrogen bonding, or mixed macromolecular systems. The crucial point here is the heterogeneous spatial distribution of carboxyl groups within the macromolecular coil, which emerges in fluctuating electrostatic fields. Locally, such fields modulate the degree of ionisation and create regions of strong charge separation, thereby generating collective interactions between coils. A modified analogue of the Poisson–Boltzmann equation derived in this work confirms that such fluctuations fundamentally determine the macroscopic properties of the solution. The results presented herein extend this knowledge even in very simple hydrophilic polymer systems and demonstrate their intrinsic neuromorphic properties and their capability to behave as minimal evolving systems in the sense of the complex-systems theory. The above insight opens new directions in the development of neuromorphic materials, organic electronics, soft metamaterials, and for reconsideration of classical data in the field of polymer–metal ion interactions. In the end, this work places polyacid solutions as promising model platforms in the study of self-organisation, information processing, and possible mechanisms relevant to prebiological evolution.

## Figures and Tables

**Table 1:** Qualitative comparison of the results reported in [28,29] with those obtained in the present study.

Characteristic	Previously Published Studies [28,29]	Present Study
Studied solution	The solution contains two types of macromolecules.	The solution contains only one type of macromolecule.
Type of interactions	Interactions occur directly between the functional groups of macromolecules (e.g., hydrophobic interactions).	Interactions between macromolecular coils are influenced by the heterogeneous distribution of ionogenic groups within the solution volume.
Nature of the theoretical model	The model is based on the analysis of the balance of forces determining the compression and extension of a macromolecular coil.	The model is based on the consideration of heterogeneous electric fields arising from fluctuations in the distribution of ionogenic groups.

## Data Availability

The original contributions presented in this study are included in the article. Further inquiries can be directed to the corresponding authors.

## Supplementary Materials

The following supporting information can be downloaded at https://www.mdpi.com/article/10.3390/polym18020279/s1.
