# Supplementary material for: Polyacid Solutions as an Analogue of a Neural Network
Source: Polymers (Basel). 2026 Jan 20;18(2):279. doi: 10.3390/polym18020279 (PMC12846287; doi:10.3390/polym18020279)
Supplement: Supplementary file 1 [file polymers-18-00279-s001.zip › polymers-4061589-supplementary.pdf]

Supplementary Materials

Finding profiles of the main reduced concentrations of positive and negative charges within a model macromolecular coil

Equation (38) for the reduced potential in the system under consideration can be rewritten in the form

$$\frac{df}{dy} = \pm\sqrt{\exp(-2f) + \exp(2f) - 2} \tag{S1}$$

where

$$f = \frac{1}{2}(\Phi - \ln Z_0^+) = \frac{1}{2}(\Phi + \ln Z_0^-);\, y = \frac{x}{\sqrt{2}\lambda} \tag{S2}$$

The transition from the reduced potential Φ to the reduced potential f, means, among other things, that the potential is now measured from the point at which the reduced concentration of positive charges reaches its maximum value (this point is located deep within the model macromolecular coil). Consequently, for the entire range of y coordinates under consideration, the following condition must be satisfied

$$f > 0 \tag{S3}$$

The subdominated expression in (S1) reduces to a perfect square, therefore

$$\mp\frac{df}{dy} = \exp(f) - \exp(-f) \tag{S4}$$

Let's make the following replacement

$$f = \ln Y \tag{S5}$$

From which

$$\mp\frac{dY}{dy} = Y^2 - 1 \tag{S6}$$

Integrating (S6), we obtain

$$\int \frac{dY}{Y^2 - 1} = \pm(y - y_0) \tag{S7}$$

The integral on the left-hand side (S7) is from integral talice, we have

$$\frac{1}{2}\ln\left|\frac{Y-1}{Y+1}\right| = \pm(y - y_0) \tag{S8}$$

Let us take into account that the sought value f is the logarithm of the value Y, and condition (S3) must also be satisfied. This means that in the case under consideration, $Y > 1$, must be satisfied, i.e.

$$\frac{Y-1}{Y+1} = \exp \pm 2(y - y_0) \tag{S9}$$

From which

$$Y = \frac{1+\exp 2(y-y_0)}{1-exp\, 2(y-y_0)} \tag{S10}$$

In this formula, the plus sign is chosen because, in accordance with the selected coordinate system (Figure 3), the area $y < 0.$ is considered. The boundary condition is chosen based on the solution to the problem corresponding to remote model coils:

$$\ln Y = \frac{1}{2} \ln Z_0^- \quad \text{(S11)}$$

From which

$$\frac{1+\exp(-2y_0)}{1-exp(-2y_0)} = \sqrt{2} \quad \text{(S12)}$$

or

$$y_0 = \frac{1}{2} \ln\left(\frac{\sqrt{2}+1}{\sqrt{2}-1}\right) \quad \text{(S13)}$$

The curves shown in Figure 4 were calculated using the following formulas obtained using expression (S10)

$$\Phi = 2f - \ln Z_0^- \quad \text{(S14)}$$

$$Z^{\pm} = Z_0^{\pm} \exp \mp\Phi \quad \text{(S15)}$$
